# Diabetes Insipidus: A Pragmatic Approach to Management

**DOI:** 10.7759/cureus.12498

**Published:** 2021-01-05

**Authors:** Gagan Priya, Sanjay Kalra, Arundhati Dasgupta, Emmy Grewal

**Affiliations:** 1 Endocrinology, Fortis Hospital, Mohali, IND; 2 Endocrinology, Bharti Research Institute of Diabetes and Endocrinology (BRIDE), Karnal, IND; 3 Endocrinology, Rudraksh Centre, Siliguri, IND; 4 Endocrinology, Max Hospital, Mohali, IND

**Keywords:** diabetes insipidus, nephrogenic diabetes insipidus, central diabetes insipidus, desmopressin

## Abstract

Diabetes insipidus (DI) is a disorder of water balance characterized by polyuria and polydipsia. It can occur due to genetic and acquired causes that affect the secretion or action of arginine vasopressin (AVP) or antidiuretic hormone (ADH).Markedly increased thirst and urination are not only quite distressing but also increases the risk of volume depletion and hypernatremia in severe situations. A careful diagnosis of the type of DI and its etiology is based on careful clinical evaluation, measurement of urine and serum osmolality, and water deprivation test. Management includes the correction of any water deficit and the use of specific pharmacological agents, including desmopressin, thiazides, and amiloride.

## Introduction and background

Diabetes insipidus (DI) is a disorder of water balance characterized by polyuria and polydipsia. It can occur at any age, and the reported prevalence is approximately 1:25,000. It can occur due to genetic (10%) and acquired (90%) causes that affect the secretion or action of arginine vasopressin (AVP) or antidiuretic hormone (ADH) [1]. 

While DI is rare in general practice, it is not so infrequent in the endocrine and neurosurgical units. Markedly increased thirst and urination are not only quite distressing but also increases the risk of volume depletion and hypernatremia in severe situations. We conducted a PubMed® search using the terms ‘diabetes insipidus’, ‘central diabetes insipidus’, ‘nephrogenic diabetes insipidus’, ‘psychogenic polydipsia’, ‘vasopressin’ and ‘desmopressin’ for this review and selected full-text of relevant articles.

## Review

Water homeostasis

Water balance is maintained by AVP, thirst mechanism, and kidneys. Daily, 170 L of urine is filtered at the glomerulus; most is reabsorbed from the renal tubules with excretion of only 1% as urine. Seventy percent of water reabsorption is from the proximal tubule through aquaporin-1 (AQP-1) channels, while the rest is reabsorbed from the loop of Henle and distal tubule by AQP-2, 3, and 4 channels [1].

Plasma osmolality is closely regulated to a narrow range of 285±5 mOsm/kg of water by AVP and thirst mechanism [1, 2]. Plasma osmolality is sensed by the osmoregulatory neurons in the hypothalamus. In the normal state, plasma AVP concentration is approximately 4 pg/ml. If plasma osmolality decreases even slightly (to 280 mOsm//kg), AVP secretion is suppressed. On the other hand, with a small increase in serum osmolality, AVP secretion increases two-fold (at 285 mOsm/kg) and thirst is also stimulated (at 290 mOsm/kg) [3].^ ^

Other stimuli that trigger AVP secretion include a decrease in circulating blood volume sensed by baroreceptors (carotid arteries, aorta, and atria), postural hypotension, nausea, acetylcholine, cholecystokinin, and certain medications [2, 4]. Apelin, a recently discovered peptide expressed in the hypothalamus, is a potent diuretic neuropeptide that inhibits AVP secretion.

AVP, in turn, regulates water homeostasis and plasma osmolality. AVP or ADH is a small peptide hormone produced by the magnocellular neurons in the supra-optic and para-ventricular nuclei of the hypothalamus that project into the posterior pituitary via the stalk. AVP is encoded by the AVP-neurophysin II gene (AVP-NPII) and is synthesized as a precursor complex that contains AVP, NPII, and copeptin. AVP reaches the posterior pituitary gland through the stalk and is then secreted into circulation.

AVP acts on vasopressin-2 receptors (V2R) in the kidney, located on the basolateral membrane of principal cells in the thick ascending limb of the loop of Henle and collecting ducts of nephrons. Resultant action through Gs-adenylyl cyclase and increased cyclic adenosine monophosphate (cAMP) levels leads to activation of protein kinase A and subsequent translocation of AQP-2 water channels on the luminal surface. Therefore, more water diffuses into the principal cells and then into interstitial tissue via the AQP-3 and 4 channels that are constitutively expressed on the basolateral membrane of these cells [4]. This results in increased water reabsorption across the renal medullary concentration gradient. As a result, kidneys can concentrate urine [3].

Diabetes insipidus

Diabetes insipidus derives its name from the defining feature of hypotonic, dilute, and insipid (tasteless) urine. The hallmarks of DI include polyuria (urine output of >40-50 ml/kg/day in adults and >100 ml/kg/day in children) with the passage of dilute urine (urine osmolality <300 mOsm/L) and polydipsia. DI occurs due to inadequate secretion of AVP or impaired response of the kidneys to AVP. Therefore, the urinary concentrating ability of the kidneys is impaired, and the nephrons pass large quantities of dilute urine, irrespective of hydration status. The clinic spectrum varies from partial to complete DI [5].

Various types of DI include central, nephrogenic, and gestational DI and the related condition of primary polydipsia (PP).

Central Diabetes Insipidus

Central DI (CDI) results from defects in synthesis, transport and/or secretion of AVP due to a wide range of causes, including both acquired and congenital, as enlisted in Table 1. Congenital CDI (10%) occurs due to mutations in the AVP-NPII gene1 [4], or wolframin (WFS1) gene (Wolfram syndrome) [3]. Wolfram or DIDMOAD syndrome is characterized by central diabetes Insipidus, diabetes mellitus, optic atrophy and sensorineural deafness.

**Table 1:** Causes of central diabetes insipidus AVP-NPII - arginine vasopressin-neurophysin II gene; ACA - anterior cerebral artery

	Causes
Congenital	Autosomal dominant – mutations in AVP-NPII gene. Autosomal recessive – Wolfram syndrome, mutations in WFS1 gene, septo-optic dysplasia, Alstrom syndrome, Hartsfield syndrome X-linked recessive
Acquired	Traumatic injury to the hypothalamus or posterior pituitary – intracranial or transsphenoidal surgery, blunt or penetrating head injury, deceleration injury. Vascular causes – intracranial hemorrhage (cerebral or hypothalamic), hypothalamic infarction, Sheehan’s syndrome, ACA aneurysm, ligation of ACA, hypoxic encephalopathy. Tumors – craniopharyngioma, pituitary macroadenoma, meningioma, germinoma, brain metastasis. Chronic granulomatous diseases – Langerhans cell histiocytosis, sarcoidosis, tubercular granulomas, neuro-sarcoidosis Infections – meningitis (bacterial, tubercular, cryptococcal), encephalitis, toxoplasmosis. Autoimmune – lymphocytic neurohypophysitis, xanthogranulomatous hypophysitis. Drugs and toxins – ethanol, snake venom, phenytoin Idiopathic.

Acquired DI results when more than 80% of AVP-secreting neurons have been damaged. The causes include penetrating or blunt head injuries and pituitary surgery. A traumatic injury can result in the direct destruction of AVP-secreting neurons or lead to ischemia and hypoxia of the hypothalamic-pituitary region as a result of vascular insult or raised intracranial pressure. The incidence of central DI after pituitary surgery varies and can be either transient or permanent. Minimally invasive procedures such as endoscopic transsphenoidal surgery (TSS) carry a lower risk of postoperative DI [6]. Another important cause of acquired CDI is intracranial tumors, infections, and infiltrative disorders. Langerhans cell histiocytosis (LCH) is a rare disorder that should be suspected in cases of CDI with multisystem (skeletal, pulmonary, dermatological, anterior pituitary dysfunction) involvement, but many patients may present with DI as the initial manifestation. Idiopathic central DI comprises almost one-fourth of cases [7]. AVP cell antibodies have been demonstrated in one-third of patients with idiopathic CDI [8].

Nephrogenic Diabetes Insipidus

Nephrogenic DI (NDI) occurs from an inability to concentrate urine in response to AVP due to congenital or acquired causes, enlisted in table 2. Complete NDI is typically associated with very high urine output of as much as 12 L daily. The commonest cause of NDI in children is inherited - 90% of cases are due to X-linked mutations in the vasopressin receptor 2 (V2R) gene; 10% cases are autosomal dominant due to mutations in the AQP-2 gene [4]. These children usually present in the first year of life with frequent wet diapers, irritability, vomiting, dehydration, and failure to thrive [9].

**Table 2:** Causes of nephrogenic diabetes insipidus V2R - vasopressin receptor 2; AQP - aquaporin

	Causes
Congenital	Mutations in V2R – X-linked recessive. Mutations in AQP-2 – autosomal recessive Bartter syndrome polyhydramnios, megaloencephaly, symptomatic epilepsy (PSME) syndrome.
Acquired	Drugs – lithium, cisplatin, vinblastine, methoxyflurane, demeclocycline, amphotericin B, aminoglycosides, colchicine. Infiltrating disorders – sarcoidosis, amyloidosis, Sjogren’s syndrome. Metabolic disorders – hypercalcemia, hypokalemia. Hematological – multiple myeloma, sickle cell anemia. Renal disorders – acute or chronic kidney disease, obstructive uropathy, polycystic kidney disease, renal infarction.

In adults, acquired causes of nephrogenic DI are more common and include electrolyte abnormalities (hypokalemia and hypercalcemia), renal, hematological and infiltrative diseases and medications. Lithium can enter the principal cells of the collecting duct via epithelial sodium channels (EnaC) and inhibits the translocation of AQP-2 channels to the cell surface. Further long-term exposure may lead to downregulation of AQP-2 gene expression. In acquired nephrogenic DI, polyuria is of moderate severity (3-4 L/day).

Gestational Diabetes Insipidus

Gestational DI is rare (2-4 per 100,000 pregnancies), occurs in the third trimester, and spontaneously resolves 4-6 weeks after delivery [10]. The cause is increased degradation of AVP by vasopressinase, expressed by the placental trophoblasts [11]. There may be a mild underlying deficiency of AVP in these women that is unmasked during pregnancy. In addition, increased prostaglandin production may also blunt the action of AVP [10]. The risk of gestational DI is higher in women with liver disease, possibly because vasopressinase is metabolized by the liver. However, gestational DI may remain undiagnosed as polyuria is considered normal in pregnancy.

Primary Polydipsia

Excessive water intake over a prolonged period of time can also result in symptoms similar to DI and is classified as primary polydipsia (PP). This may occur as a result of an abnormal thirst mechanism or increased thirst (dipsogenic DI). Dipsogenic DI may occur due to chronic meningitis, chronic granulomatous diseases such as sarcoidosis, multiple sclerosis, tubercular meningitis, hypothalamic tumors, or injury.

Some patients may have underlying psychiatric illnesses such as compulsive disorders or schizophrenia and are classified as psychogenic polydipsia. Dry mouth may also result from certain drugs such as phenothiazines or anticholinergic agents. Due to excess fluid intake, serum osmolality is reduced and AVP secretion is suppressed in PP.

Clinical Presentation

The most common presenting symptom of DI is significant polyuria, defined as urine output of >40-50 ml/kg/day (at least 2.5-3 L/day, but may exceed 8-16 L) and polydipsia, defined as water intake of > 100 ml/kg/day [5, 12]. Polyuria, however, should be differentiated from frequent micturition, urgency or dysuria, and other causes of polyuria as enlisted in Table 3.

**Table 3:** Differential diagnosis of polyuria AVP - arginine vasopressin; SGLT2 - sodium-glucose co-transporter 2

Category	Causes	Diagnostic clues
Frequency or urgency of micturition	Urinary infection; prostate hyperplasia; urinary incontinence; bladder dysfunction	Patient does not have actual polyuria, urine output < 40 ml/kg/day.
Osmotic diuresis	Glucose – diabetes, stress hyperglycemia, SGLT2 inhibitors. Urea – high protein intake, steroids, catabolic state, recovery from acute renal failure. Medications – diuretics, mannitol. Sodium – large amounts of IV saline, post-obstructive uropathy.	Clinical background – diabetes mellitus, medications, obstructive uropathy, renal failure, catabolic state, intravenous fluids given for resuscitation. Polyuria present, urine osmolality is high. Urine examination positive for glucose, renal dysfunction may be present.
Primary Polydipsia	Increased intake of water due to abnormal thirst. Compulsive fluid consumption – psychogenic polydipsia	History of psychiatric illness. Water deprivation test – individual concentrates urine with an increase in urine osmolality.
Diabetes insipidus	Central – inadequate secretion of AVP. Nephrogenic – impaired response of the kidneys to AVP. Gestational – increased degradation of AVP by vasopressinase.	Urine osmolality is low, plasma osmolality and serum sodium may be elevated. Diagnosis made by water deprivation test.

Onset may be gradual or abrupt depending on the cause. Polyuria and polydipsia can result in significant distress. Individuals may complain of nocturia, disturbed sleep, fatigue, postural dizziness, and weight loss. Dehydration occurs only in severe cases, especially if there is a lack of access to water (as in infants or an unconscious patient) or thirst mechanism is impaired. In children, symptoms range from frequent urination, nocturia, enuresis, constant thirst, and irritability. Severe cases are at risk of dehydration, growth retardation, and even failure to thrive. On examination, there may be signs of dehydration such as dry mucosa, loss of skin turgor, hypotension, or tachycardia. Severe dehydration along with electrolyte disturbances, may rarely result in altered sensorium.

History and physical examination should also focus on features that may suggest intracranial disease, head trauma or surgery, anterior pituitary hormone deficits, medications, kidney disease, or systemic illness. Psychogenic polydipsia should be suspected in individuals with gradual onset of symptoms, fluctuating symptoms, and a history of psychiatric illness.

Adipsic Diabetes

Adipsic diabetes is characterized by impaired thirst despite a rise in serum osmolality and may result from a variety of intracranial causes that result in damage to the anterior hypothalamus, such as brain injury, ACA aneurysm, surgery for craniopharyngioma, or neurosarcoidosis [7]. These individuals are at high risk of dehydration and life-threatening hypernatremia, which can cause altered sensorium, seizures, and intracranial bleed [1].

Evaluation and diagnosis

The evaluation of suspected DI includes the following steps:

1. Confirmation of polyuria,

2. Confirmation of hypotonic polyuria,

3. Determining the type of DI,

4. Determining the etiology of DI.

The first step is the confirmation of actual polyuria, defined as urine output of ≥40-50 ml/kg/day or 3 L/day. In case of doubt, an estimation of 24-hour urine volume is useful. If polyuria is confirmed, urine specific gravity and urine osmolality is measured to determine if the person has hypotonic polyuria. In DI, urine osmolality is <300 mOsm/kg. Urine specific gravity <1.010 can be used for rapid screening since the results of urine osmolality may not be immediately available. Urine osmolality >800 mOsm/kg indicates solute diuresis and requires evaluation for other causes such as diabetes mellitus, kidney disease, medications (diuretics, mannitol). Table 3 provides the differential diagnosis of polyuria.

Serum electrolytes and osmolality, calcium and renal functions are also useful. Serum osmolality is calculated from serum sodium, blood glucose, and urea [5]:

Ø Plasma osmolality = 2 [Na+] + [blood glucose/18] + [blood urea nitrogen/2.8]

If hypotonic polyuria is documented, a careful evaluation of the type and cause of DI is needed. The presence of urine hypoosmolality (<300 mOsm/kg) and plasma hyperosmolality (>300 mOsm/kg) with polyuria clinches the diagnosis of DI. Hypernatremia (Na >145 mEq/L) and high serum osmolality (>300 mOsm/kg) suggest DI, while both remain normal or low in PP [2]. However, many individuals have indeterminate urine osmolality (300-800 mOsm/kg) and normal serum osmolality and electrolytes, requiring further confirmatory tests. This would require dynamic tests of the AVP-kidney axis (water deprivation test or infusion of hypertonic saline) and the estimation of plasma ADH concentration [7]. Misdiagnosis and inappropriate treatment carry significant risks, e.g., if desmopressin is used for primary polydipsia, it can cause hyponatremia.

Water deprivation test

**Figure 1:**
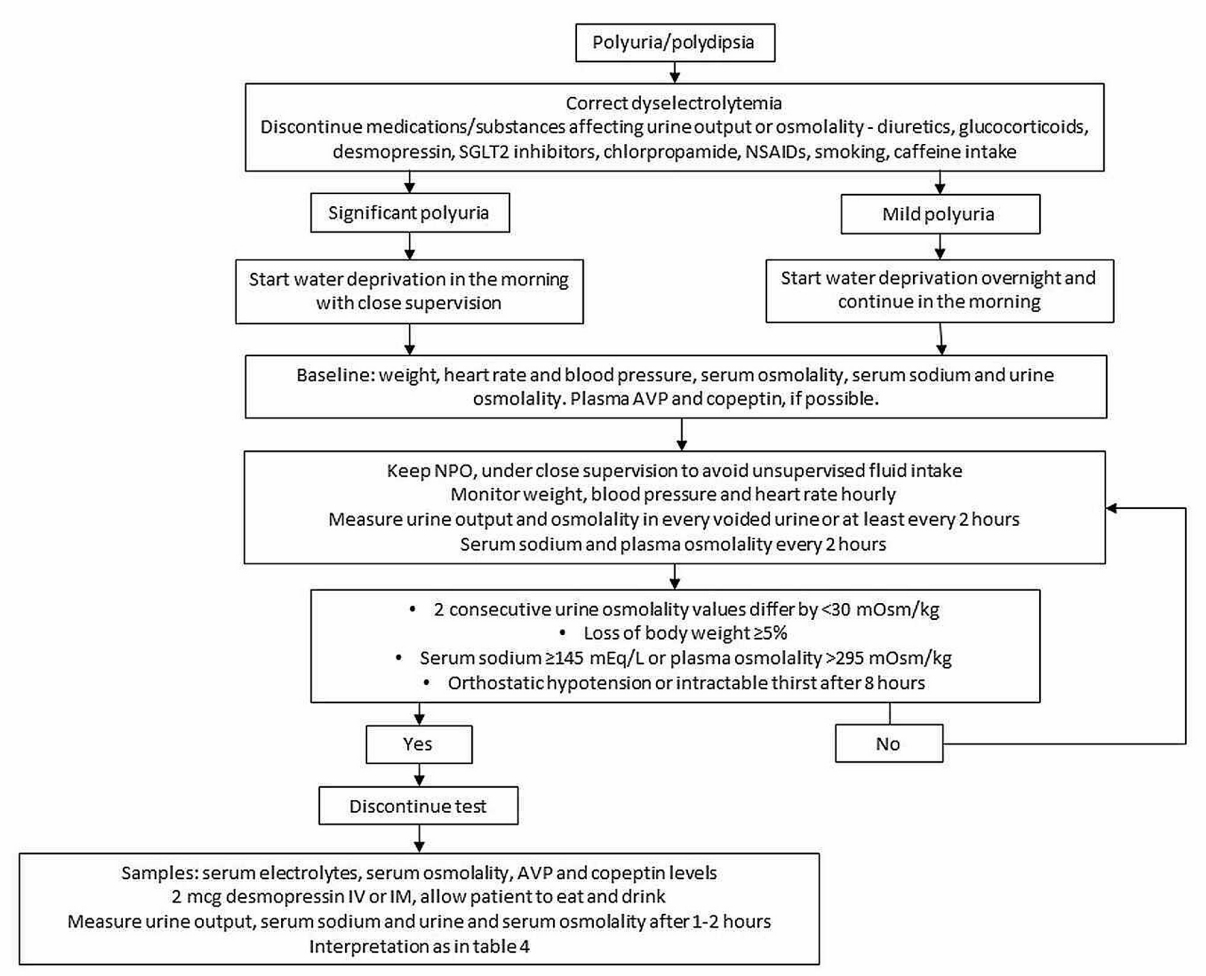
Protocol for water deprivation test AVP - arginine vasopressin; NSAIDs - non-steroidal anti-inflammatory drugs; SGLT2 - sodium glucose transporter 2

Water deprivation test is an important test in the evaluation of suspected DI [13]. In response to water deprivation, AVP secretion is increased in normal individuals and those with PP, leading to a rise in urine osmolality >800 mOsm/kg. On the other hand, in CDI or NDI, there is an inability to concentrate urine in response to water deprivation and urine remains dilute (urine osmolality <300 mOsm/kg). After the administration of desmopressin, urine osmolality rises (>800 mOsm/kg) in CDI but not in NDI. The test protocol is described in Figure 1 and interpreted in Table 4. However, a significant proportion of patients may demonstrate urine osmolality of 300-800 mOsm/kg following water deprivation and do not exhibit a robust response to AVP. These include patients with partial DI (central or nephrogenic) and long-standing PP (in which renal medullary concentration gradient is reduced, mimicking nephrogenic DI) [14].

**Table 4:** Interpretation of water deprivation test AVP - arginine vasopressin; DI - diabetes insipidus

	Normal	Central DI	Nephrogenic DI	Primary polydipsia	Partial DI
Baseline urine osmolality (mOsm/kg)	>300	<300	<300	300-800	300-800
Urine osmolality (mOsm/kg) after water deprivation	800-1200	<300	<300	300-800	300-800
Plasma osmolality after water deprivation	Normal	Increases	Increases	Normal	Normal
Serum sodium (mEq/L)	Normal	May increase (>145)	May increase (>145)	Normal	Normal
Urine osmolality after administration of desmopressin	-	Increases >50%	Does not increase	-	<50 % increase
Baseline serum AVP and copeptin	Normal	Low	Increased	Normal	Normal to low

Measurement of AVP and copeptin

AVP levels are measured at the end of the water deprivation test (before administering AVP). AVP levels are low in CDI, elevated NDI, and may be normal or low in long-standing PP and partial CDI. However, since AVP is a small peptide that is rapidly cleared, its estimation is difficult and seldom done. Moreover, it is bound to platelets through vasopressin 1 (V1) receptors and levels may vary widely if the sample is not processed immediately and stored at -70°C.

Copeptin (C-terminal peptide of pro-vasopressin) is co-secreted with AVP and can be a surrogate of AVP secretion as it is more stable [15]. Baseline copeptin <2.6 pmol/L is diagnostic of CDI, while levels >21.4 pmol/L are diagnostic of NDI. If baseline levels are indeterminate, it could be either CDI or PP and copeptin is estimated after hypertonic saline infusion. A level of >4.9 pmol/L suggests PP [7].

Hypertonic saline infusion test

**Figure 2:**
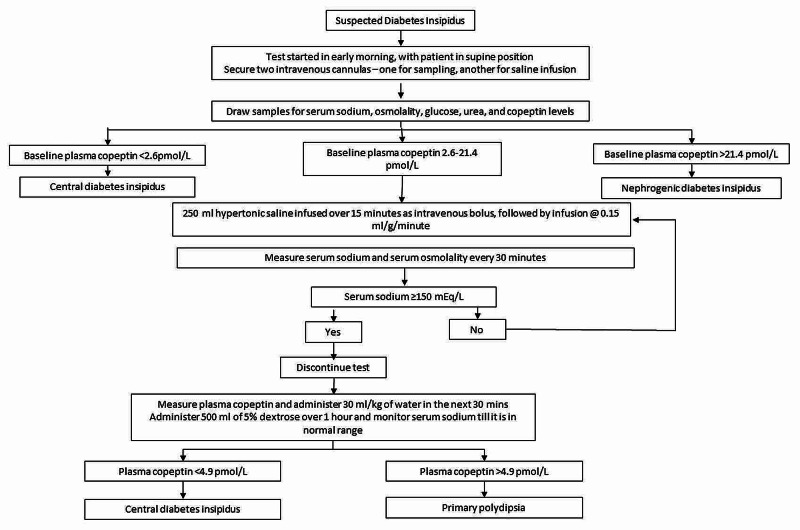
Protocol of hypertonic saline infusion test

Hypertonic saline infusion along with measurement of copeptin can be considered if results of water deprivation are inconclusive. Figure 2 provides an algorithm for performing the hypertonic saline infusion test. Baseline plasma copeptin is >21.4 pmol/l in NDI while levels <2.6 pmol/L indicate CDI. Following hypertonic saline infusion, plasma copeptin levels <4.9 pmol/L suggest CDI, while levels of >4.9 pmol/L indicate PP [15].

Other evaluation

In doubtful cases, a careful therapeutic trial of desmopressin can be considered. Desmopressin 100 mcg is given orally every 12 hours for 48 hours; if symptoms resolve, it suggests CDI or PP; while there is no response in NDI. However, care must be taken as hyponatremia can occur in individuals with PP [16].

Further testing should be guided by clinical suspicion to determine the cause of DI [1]. On magnetic resonance imaging (MRI) of the sella and hypothalamus, the absence of posterior pituitary bright spot (on T1 weighted images) or thickened stalk (>3 mm) suggests CDI [1, 17]. On the other hand, PP is characterized by normal posterior pituitary bright spot and pituitary stalk. MRI may also be useful in the detection of other causes of CDI as enlisted in Table 1.

In cases of CDI, evaluation of anterior pituitary functions is warranted as many individuals may have anterior pituitary hormone deficits, including growth hormone deficiency, partial or complete hypopituitarism [17]. Adrenal insufficiency may mask central DI that becomes apparent only after glucocorticoid replacement.

**Figure 3:**
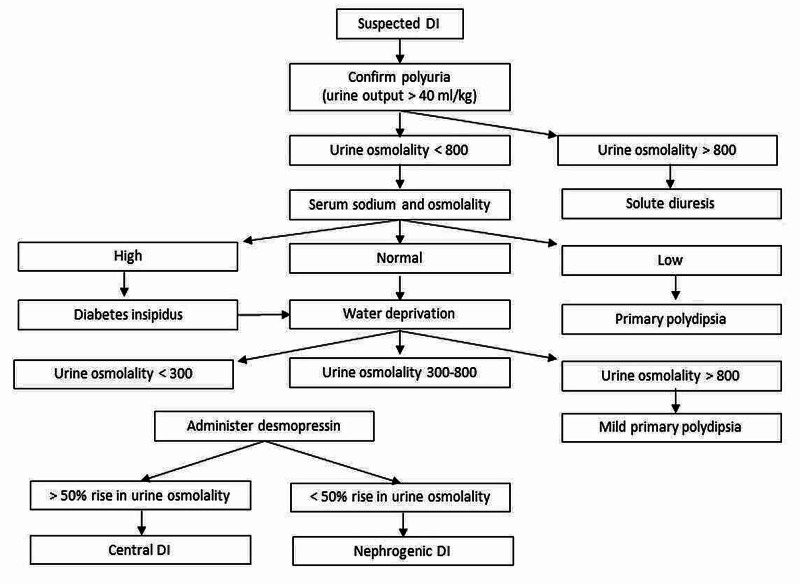
Approach to polyuria evaluation DI - diabetes insipidus

Figure 3 provides an algorithm for the approach to polyuria evaluation.

Management of diabetes insipidus

The first concern is adequate hydration and replacement of water deficit, which is calculated as [6]:

Water deficit (liters) = 0.6 x body weight (kg) x [(serum Na/140) - 1] ​​​​​​

In most individuals who have normal thirst mechanism, it can guide the intake of oral fluids. However, in individuals with adipsic DI who have impaired thirst, a daily fluid intake should be fixed at which euvolemia and eunatremia are maintained [1]. They will also require more frequent monitoring of weight, intake/output records, and electrolytes.

In unconscious patients, water deficit can be corrected with plain water administered through Ryle’s tube and with intravenous hypotonic fluids (5% dextrose or 0.45 saline) [3]. Isotonic fluid (0.9 saline) should be avoided as it can worsen hypernatremia. In NDI due to kidney disease, reduction of osmotic load by restricting the intake of sodium and proteins is important since increased solute excretion contributes to further increased urine output [9]. Causative agents, such as lithium should be discontinued.

In mild DI, fluid replacement is adequate, but more severe cases require pharmacological treatment [3]. Pharmacological management depends on the underlying cause. Most cases of CDI can be effectively managed with vasopressin or its analogue, desmopressin. Treatment of the underlying cause of CDI and NDI is also important. As follows, we describe medications used in the management of DI.

Desmopressin

Posterior pituitary extracts that contained vasopressin and oxytocin were used for the management of DI in the early 1900s. Pitressin (vasopressin tannate in oil) later became widely available but had significant vasopressor effects. Desmopressin or 1-deamino-8-D-AVP (DDAVP), a synthetic analogue of vasopressin, became available in the 1970s [12]. Desmopressin is resistant to degradation by vasopressinase and has a greater anti-diuretic effect with 2000-fold lower vasopressor activity [1]. So, it is associated with less risk of side effects such as vasoconstriction or hyponatremia.

Several formulations of desmopressin are available as enlisted in Table 5. With oral or nasal formulations, the maximum drug concentration is achieved within one hour; a decrease in urine output occurs in 1-2 hours and the effect lasts 6-18 hours [12].^ ^Individual variations in clinical response exist. Therefore, the dose has to be adjusted at a weekly interval till a stable dose is attained over one month.

**Table 5:** Various formulations of desmopressin

Formulation	Dose	Advantages	Disadvantages
Intranasal spray	10-40 mcg (1 puff is 10 mcg)	More rapid onset and longer duration of action.	Need storage in cold chain for stability. Absorption may be erratic and impaired if there is nasal congestion, nasal discharge or chronic rhinitis.
Oral tablet	100-400 mcg (tablets of 100 mcg)	Easy to administer. Absorbed unaltered from the gastrointestinal tract. As effective as nasal sprays. Less risk of hyponatremia than nasal sprays.	
Oral disintegrating tablet	Same as oral tablet	Dissolves instantly in the mouth – 60% greater bioavailability. Better quality of life.	Not easily available. Higher cost.
Injectable	4 mcg (maximum 0.4 mcg/kg) 4 mcg/ml injections	Intravenous, intramuscular or subcutaneous use. Used in the water deprivation test and if nasal or oral administration is not possible.	Robust effect – more risk of water intoxication or hyponatremia.

Safety concerns: The most common adverse effects are hyponatremia and water intoxication, though the risk remains low [3]. No effect on blood pressure, heart rate, or body weight has been demonstrated and desmopressin appears to have no major safety concerns. Desmopressin also appears safe during pregnancy for gestational DI.

Other Drugs

Several other medications, including thiazide diuretics, carbamazepine, indomethacin, amiloride, etc., are available for the management of DI, especially NDI. The mechanism of benefit and the potential role of these agents is discussed in Table 6.

**Table 6:** Other medications for the management of diabetes insipidus AQP - aquaporin 2; AVP - arginine vasopressin; CDI - central diabetes insipidus; NDI - nephrogenic diabetes insipidus; V2R - vasopressin-2 receptor

Drug	Mechanism of benefit	Potential role in diabetes insipidus
Thiazide diuretics	Inhibit sodium-chloride co-transport in distal tubule to increased urine sodium and osmolality. This results in reduced intravascular volume, increased renin-angiotensin-aldosterone activity and reduction in glomerular filtration. As a result, sodium and water reabsorption in the proximal tubule increases, reducing urine output.	Used in NDI - can reduce the urine output by almost 70% when used with a low-solute diet. Can also be used in CDI. Hydrochlorothiazide (1-2 mg/kg/day) or chlorothiazide (5-10 mg/kg/day). Caution – the risk of hypovolemia and hypokalemia.
Carbamazepine	Increases endogenous secretion of AVP. Direct action on the renal collecting ducts – increases expression of AQP-2 channels.	Can be used doses of 200-800 mg/day for both CDI and NDI.
Indapamide	Mechanism is similar to thiazides.	2-5 mg/day in mild CDI.
Amiloride	Inhibits epithelial sodium channels. Decreases transcellular lithium transport and blocks the effect of lithium on AQP-2 channel expression.	Useful in lithium-induced NDI. Combined with thiazides, it reduces the risk of hypokalemia and metabolic alkalosis.
Indomethacin	Inhibits prostaglandin synthesis.	Used in NDI, especially if not responsive to desmopressin, thiazides and amiloride. High doses may cause side effects – gastrointestinal bleeding, nephrotoxicity.
Chlorpropamide	First-generation sulfonylurea. Increases AVP secretion, also potentiates the effect of AVP.	125-1000 mg/day have been used in CDI. Risk of significant hypoglycemia, not currently used.
Clofibrate	Stimulates AVP secretion.	500 mg 6 hourly. Can cause myopathy.
Molecular chaperones	Molecular chaperones that stabilize V2R or stimulate V2R-independent AQP-2 cell membrane translocation.	Under evaluation, may be useful in NDI.

Treatment of nephrogenic DI is usually with fluid replacement, reduced solute load, thiazides and indomethacin. The addition of amiloride further reduces urine volume and the risk of hypokalemia with thiazides. Patients with partial nephrogenic DI may be responsive to desmopressin [4]. Adrenal insufficiency may mark features of partial DI that becomes apparent only after glucocorticoid replacement. Therefore, vigilance is required in patients with hypopituitarism if DI is not apparent initially.

Management of postoperative diabetes insipidus

In the postoperative period, DI should be suspected if there is polyuria (urine output >40-50 ml/kg/day or >250 ml/hour for two consecutive hours) with or without hypovolemia. On laboratory evaluation, urine specific gravity <1.010 is helpful for prompt action since the reports of electrolytes and osmolality may be delayed. Urine osmolality is usually <100 mOsm/kg; additionally, the patient may have increased serum sodium and plasma osmolality. Other causes of postoperative polyuria need to be excluded: excessive perioperative fluids, uncontrolled diabetes mellitus or stress hyperglycemia, and diuretics. Water deprivation test is not feasible in this setting. Daily monitoring of fluid intake and output and body weight and twice-daily monitoring of serum electrolytes and urine osmolality is warranted [6].

In most individuals, postoperative DI is mild and transient. Some patients may have transient CDI over 1-4 days, followed by a phase of oliguria (4-7 days) due to the release of stored AVP from degenerating neurons and then permanent DI with the destruction of the AVP-secreting neurons [6]. This calls for close monitoring of water balance and electrolytes in the immediate postoperative period.

A single dose of subcutaneous or intravenous desmopressin can be administered. The next dose is administered only if urine output begins to increase again (>250 ml/hour for two hours with low urine specific gravity and/or osmolality) to avoid water intoxication and hyponatremia [6].

## Conclusions

Diabetes insipidus is a relatively rare endocrine disorder that presents with hypotonic polyuria and polydipsia. It can result from either inadequate secretion of AVP (CDI), lack of renal response to AVP (NDI), or excessive fluid intake (PP). A careful diagnosis of the type of DI and its etiology is based on careful clinical evaluation, measurement of urine and serum osmolality, and water deprivation test. Management includes the correction of any water deficit and the use of specific pharmacological agents including desmopressin, thiazides and amiloride.

## References

[1] Di Iorgi N, Napoli F, Allegri AEM (2012). Diabetes insipidus - diagnosis and management. Horm Res Paediatr.

[2] Muhsin SA, Mount DB (2016). Diagnosis and treatment of hypernatremia. Best Pract Res Clin Endocrinol Metab.

[3] Dabrowski E, Kadakia R, Zimmerman D (2016). Diabetes insipidus in infants and children. Best Pract Res Clin Endocrinol Metab.

[4] Schernthaner-Reiter MH, Stratakis CA, Luger A (2017). Genetics of diabetes insipidus. Endocrinol Metab Clin N Am.

[5] Sarma KV (2013). Algorithmic approach for the diagnosis of polyuria. Medicine Update.

[6] Lamas C, del Pozo C, Villabona C (2014). Clinical guidelines for the management of diabetes insipidus and syndrome of inappropriate antidiuretic hormone secretion after pituitary surgery. Endocrinol Nutr.

[7] Garrahy A, Moran C, Thompson CJ (2019). Diagnosis and management of central diabetes insipidus in adults. Clin Endocrinol.

[8] Gut P, Czarnywojtek A, Ziemnicka K (2018). Incidence of pituitary autoantibodies in idiopathic diabetes insipidus. Cent Eur J Immunol.

[9] Kavanagh C, Uy NS (2019). Nephrogenic diabetes insipidus. Pediatr Clin N Am.

[10] Aleksandrov N, Audibert F, Bedard MJ (2010). Gestational diabetes insipidus: a review of an underdiagnosed condition. J Obstet Gynaecol Can.

[11] Marques P, Gunawardana K, Grossman A (2015). Transient diabetes insipidus in pregnancy. Endocrinol Diabetes Metab Case Rep.

[12] Kalra S, Zargar AH, Jain SM (2016). Diabetes insipidus: the other diabetes. Indian J Endocr Metab.

[13] Pedrosa W, Drummond JB, Soares BS, Ribeiro-Oliveira A (2018). A combined outpatient and inpatient overnight water deprivation test is effective and safe in diagnosing patients with polyuria-polydipsia syndrome. Endocrine Practice.

[14] Trimpou P, Olsson D S, Ehn O, Ragnarsson O (2017). Diagnostic value of the water deprivation test in the polyuria-polydipsia syndrome. Hormones.

[15] Christ-Crain M, Fenske W (2016). Copeptin in the diagnosis of vasopressin-dependent disorders of fluid homeostasis. Nat Rev Endocrinol.

[16] Odeh M, Oliven A (2001). Coma and seizures due to severe hyponatremia and water intoxication in an adult with intranasal desmopressin therapy for nocturnal enuresis. J Clin Pharmacol.

[17] Liu W, Hou J, Liu X, Wang L, Li G (2019). Causes and follow-up of central diabetes insipidus in children. Int J Endocrinol.

